# The Intersection of Atrial Fibrillation and Coronary Artery Disease in Middle Eastern Patients. Analysis from the Jordan Atrial Fibrillation Study

**DOI:** 10.5334/gh.1312

**Published:** 2024-03-12

**Authors:** Ayman Hammoudeh, Yahya Badaineh, Ramzi Tabbalat, Anas Ahmad, Mohammad Bahhour, Darya Ja’ara, Joud Shehadeh, Mohammad A. Jum’ah, Afnan Migdad, Mohammad Hani, Imad A. Alhaddad

**Affiliations:** 1 Department of Cardiology, Istishari Hospital, 44 Kindi Street, Amman 11954, Jordan; 2 Department of Cardiology, Abdali Hospital, 1 Istethmar Street/Abdali Boulevard, Amman 11190, Jordan; 3 Coronary Care Unit, Istishari Hospital, 44 Kindi Street, Amman 11954, Jordan; 4 Department of Internal Medicine, Istishari hospital, 44 Kindi Street, Amman 11954, Jordan; 5 Jordan Cardiovascular Center, Jordan Hospital, 4 Queen Rania Hospital, Amman, Jordan

**Keywords:** Atrial fibrillation, Coronary artery disease, Oral anticoagulant agents, Middle Eastern population, Prognosis

## Abstract

**Background:**

There is a scarcity of clinical studies which evaluate the association of atrial fibrillation (AF) and coronary artery disease (CAD) in the Middle East. The aim of this study was to evaluate the impact of CAD on baseline clinical profiles and one-year outcomes in a Middle Eastern cohort with AF.

**Methods:**

Consecutive AF patients evaluated in 29 hospitals and cardiology clinics were enrolled in the Jordan AF Study (May 2019–December 2020). Clinical and echocardiographic features, use of medications and one-year outcomes in patients with AF/CAD were compared to AF/no CAD patients.

**Results:**

Of 2020 AF patients enrolled, 216 (10.7%) had CAD. Patients with AF/CAD were more likely to be men and had significantly higher prevalence of hypertension, diabetes, dyslipidemia, heart failure and chronic kidney disease compared to the AF/no CAD patients. They also had lower mean left ventricular ejection fraction and larger left atrial size. Mean CHA_2_DS_2_ VASc and HAS-BLED scores were higher in AF/CAD patients than those with AF/no CAD (4.3 ± 1.7 vs. 3.6 ± 1.8, p < 0.0001) and (2.0 ± 1.1 vs. 1.6 ± 1.1, p < 0.0001), respectively. Oral anticoagulant agents were used in similar rates in the two groups (83.8% vs. 82.9%, p = 0.81), but more patients with AF/CAD were prescribed additional antiplatelet agents compared to patients with AF/no CAD (73.7% vs. 41.5%, p < 0.0001). At one year, AF/CAD patients, compared to AF/no CAD patients had significantly higher hospitalization rate (39.4% vs. 29.2%, p = 0.003), more acute coronary syndrome and coronary revascularization (6.9% vs. 2.4%, p = 0.004), and higher all-cause mortality (18.5% vs. 10.9%, p = 0.002).

**Conclusions:**

In this cohort of Middle Eastern patients with AF, one in 10 patients had CAD. The coexistence of AF and CAD was associated with a worse baseline clinical profile and one-year outcomes. Clinical study registration: the study is registered on clinicaltrials.gov (unique identifier number NCT03917992).

## Introduction

Atrial fibrillation (AF) is the most common sustained arrhythmia in the general population, and the incidence of AF has multiplied four to fivefold in the past half century [1]. Coronary artery disease (CAD) is the most common cardiovascular disease and is the leading cause of death in both the developed and developing countries [1,2]. Many studies have shown that AF and CAD can aggravate each other in a vicious circle and that both diseases share common cardiovascular risk factors including hypertension, diabetes mellitus, sleep apnea and obesity. The prevalence of CAD in patients with AF varies between 17% and 47%, and conversely the prevalence of AF among patients with CAD is relatively low and ranges between 0.2% and 5% [1,2,3].

The longevity of patients who have coexisting AF and CAD, especially patients with acute myocardial infarction, is significantly threatened by higher risk of stroke, heart failure, acute coronary syndrome (ACS), hospital admissions and deaths for cardiovascular causes [4,5,6]. Furthermore, management of patients with AF and CAD remains challenging and mandates a balance between minimizing the inherent risk of thromboembolic complications (stroke and systemic embolism [SSE]) of AF, the ischemic complications of CAD and the excess risk of bleeding events related to the utilization of dual pathways inhibition of the coagulation cascade by the oral anticoagulant agents (OACs) and platelet aggregation by a short- or long-term use of dual antiplatelet agents [7,8].

The prevalence of both AF and CAD is rising in the Middle East (ME), especially among younger age groups, resulting in a significant negative impact on the health of affected individuals and the community at large [9]. This rise is mainly due to improvement in life expectancy and the escalating prevalence in certain cardiovascular risk factors including diabetes mellitus, cigarette smoking and obesity in the region [10]. The clinical profiles and impact on prognosis of the coexistence of AF and CAD in ME patients have not been studied. Published studies from this region had indicated that CAD is present in 5% to 33% of patients with AF [11,12,13,14]. None of the published studies, however, systemically assessed the demographic, clinical and echocardiographic profiles and the long-term outcome in the high-risk subgroup of AF patients with a coexisting CAD.

The Jordan AF (JoFib) Study is a multicenter registry that enrolled a cohort of ME patients with valvular AF (VAF) and nonvalvular AF (NVAF) and followed them for one year. The baseline features of the studied population, adherence to the use of oral anticoagulant agents (OACs) and the one-year outcome in the whole cohort were published earlier [15,16]. The current analysis presents an analysis of baseline clinical and echocardiographic profiles, pharmacotherapy and one-year incidence of major adverse cardiovascular events in AF patients who have CAD compared to those who do not have CAD.

## Methods

The methods and baseline data from the JoFib have been described previously [16]. Briefly, the study involved consecutive AF patients (>18 years of age) evaluated at 29 hospitals and ambulatory care cardiology clinics in Jordan in the period from May 2019 through October 2020. Atrial fibrillation was diagnosed based on a 12-lead electrocardiogram (ECG), a more than 30-second rhythm strip, more than one episode of AF on ambulatory ECG monitor, or a prior AF diagnosis by a treating physician. Valvular AF included patients who have at least moderate rheumatic mitral stenosis, or heart prosthetic mechanical valve. Other patients were classified as having NVAF. Coronary artery disease was defined as a history of acute coronary syndrome (ACS) or percutaneous or surgical coronary revascularization, or the presence of significant coronary artery atherosclerotic lesions (≥50% stenosis in the left main coronary artery or ≥70% stenosis in at least one of the other epicardial coronary arteries by coronary computed tomography angiography or invasive coronary angiography. One-year follow-up was achieved through outpatient clinic visits, hospital readmission or phone calls at 1, 6 and 12 months after enrollment to document the incidence of prespecified endpoints of all-cause death, cardiovascular death, SSE, ACS, all-cause hospitalization, hospitalization for cardiovascular causes, major bleeding events and clinically relevant non-major (CRNM) bleeding. All-cause deaths included death from non-cardiovascular and cardiovascular causes. The latter included deaths due to fatal myocardial infarction, sudden cardiac death and heart failure. Stroke was diagnosed based on neurologist evaluation and standard clinical and imaging criteria. SE was diagnosed based on documented clinical, angiographic, intra-operative, or pathological evidence of thromboembolism to an arterial bed of the extremities or abdominal aorta branches. The CHA_2_DS_2_-VASc and HAS-BLED scores were calculated based on published clinical practice guidelines [17,18]. Major bleeding event included fatal bleeding, symptomatic bleeding in a critical area or organ (i.e., intracranial, intraspinal, intraocular, retroperitoneal, intraarticular or pericardial) and/or bleeding that causes a fall in hemoglobin level of >2 g/dL, or requires >2 units of whole blood or red cells transfusion, based on the International Society of Thrombosis and Hemostasis criteria [19]. Clinically relevant non-major bleeding events were those which necessitated medical and/or surgical evaluation or intervention, or required change in antithrombotic regimen [19]. Event rates in patients with AF and CAD were compared to those in patients with AF and no CAD. Institutional Review Boards of the participating centers approved the study. Each patient signed a written informed consent. The study was registered at clinicaltrials.gov (unique identifier number NCT03917992).

## Statistical analysis

Descriptive statistics were performed using means and standard deviation (SD) to describe the continuous variables and percentages were used to describe the categorical variables. Independent t-test was used to compare means and chi-square test was used to compare percentages of the variables in AF patients who had CAD and those who did not have CAD. P-value of <0.05 was considered statistically significant.

## Results

A total of 2020 patients with AF were enrolled, including 216 (10.7%) who had CAD. The majority of patients with CAD (206, 95.4%) were diagnosed to have CAD prior to enrollment in the study and only 10 patients (4.6%) were diagnosed with ACS upon admission to the study. Table 1 depicts the demographic, clinical and echocardiographic profiles of the AF/CAD and AF/no CAD groups. Patients in the AF/CAD group were more likely to be men, had significantly higher prevalence of hypertension, diabetes mellitus and dyslipidemia, and higher mean CHA_2_DS_2_-VASc and HAS-BLED scores compared to the AF/no CAD group. They also had higher prevalence of clinically important comorbid disease, including heart failure and chronic kidney disease, and were more likely to be enrolled in an in-patient, rather than ambulatory setting. Of the four echocardiographic features evaluated in both groups, patients with AF/CAD had lower mean left ventricular ejection fraction and larger left atrial diameter than patients in the AF/no CAD group.

**Table 1:** Baseline demographic, clinical and echocardiography characteristics of 2020 AF patients with or without coronary artery disease.


CLINICAL FEATURE	AF AND CAD N = 216 (10.7%)	AF AND NO CAD N = 1804 (89.3%)	P-VALUE

Age (years), mean + SD	69.3 ± 10.6	67.7 ± 13.1	0.08

Women, N (%)	81 (37.5)	1014 (56.2)	<0.0001

Hypertension, N (%)	182 (84.3)	1324 (73.4)	0.007

Diabetes mellitus, N (%)	118 (54.6)	763 (42.3)	0.008

Dyslipidemia, N (%)	145 (67.1)	764 (42.4)	<0.0001

Current cigarette smoking, N (%)	38 (17.6)	242 (13.4)	0.11

Enrolled in an outpatient setting, N (%)	92 (42.6)	464 (25.7)	<0.0001

Non-paroxysmal AF	139 (64.4)	1153 (63.9)	0.94

Classification of AF:			0.04

- Valvular AF	10 (4.6)	160 (8.9)	

- Nonvalvular AF	206 (95.4)	1644 (91.1)	

Past Stroke/SE, N (%)	32 (14.8)	310 (17.2)	0.43

CKD, N (%)	26 (12.0)	155 (8.6)	<0.0001

Sleep apnea, N (%)	9 (4.2)	76 (4.2)	0.86

Heart failure, N (%)	64 (29.6)	416 (23.1)	0.04

HAS-BLED score, mean ± SD	2.0 ± 1.1	1.6 ± 1.1	<0.0001

CHA_2_DS_2_-VASc score, mean ± SD	4.3 ± 1.7	3.6 ± 1.8	<0.0001

Echocardiographic features*:			

- LVEF (%) mean + SD	49.2 + 13.4	53.6 + 12.8	<0.0001

- LVEF ≤ 40%, %	25.9	16.9	0.002

- LA diameter ≥ 4.5 cm, %	64.9	42.8	<0.0001

- LVH, %	28.4	40.1	0.002

- Pulmonary hypertension, %	21.8	58.2	<0.0001


AF: atrial fibrillation; CAD: coronary artery disease; CKD: chronic kidney disease; LA: left atrial; LVEF: left ventricular ejection fraction; LVH: left ventricular hypertrophy; SE: systemic embolism. *Transthoracic echocardiography was done for 93% of patients.

The majority of patients in the whole cohort were prescribed OACs with no significant differences between both groups of AF/CAD and AF/no CAD (Table 2), but direct OACs (DOACs) were more used than vitamin K antagonists (VKA) in patients with AF/CAD then in those with AF/no CAD. Furthermore, the use of one or two oral antiplatelet agents, overall and in combination with OACs, was higher in the AF/CAD group. More patients in the AF/CAD group were prescribed the three major cardiovascular medications (beta blockers, renin-angiotensin system blockers and statins).

**Table 2:** Use of oral anticoagulant and antiplatelet agents, and other cardiovascular medications in AF patients with and without coronary artery disease.


MEDICATIONS	AF AND CAD N = 216 (10.7%)	AF AND NO CAD N = 1804 (89.3%)	P-VALUE

Oral anticoagulant agents, N (%)			

- All OACs	181 (83.8)	1496 (82.9)	0.81

- VKA	56 (25.9)	606 (33.6)	0.03

- DOACs	125 (57.9)	890 (49.3)	0.02

Oral antiplatelet agents, N (%)			

Single agent	120 (55.6)	670 (37.1)	<0.0001

Dual agents	39 (18.1)	85 (4.7)	

Single or dual agents	159 (73.6)	755 (41.9)	

OACs and antiplatelet combination, N (%)			

OAC and one or two antiplatelet agents	121 (56.0)	527 (29.2)	<0.0001

Oral antiarrhythmic agents and other medications, N (%)			

Amiodarone	41 (19.0)	346 (19.2)	0.98

Class I antiarrhythmic medications	2 (0.9)	35 (1.9)	0.44

Beta blockers	185 (85.6)	1434 (79.5)	0.04

Digitalis	20 (9.3)	299 (16.6)	0.007

Non-dihydropyridine CCB	13 (6.0)	206 (11.4)	0.02

RAS inhibitors	106 (49.1)	675 (37.4)	0.001

Statins	149 (69.0)	603 (33.4)	<0.0001


AF: atrial fibrillation; CAD: coronary artery disease; CCB: calcium channel blocker; DOACs: direct oral anticoagulant agents, OACs: oral anticoagulant agents; RAS: renin angiotensin system; VKA: vitamin K antagonists.

**Figure 1:**
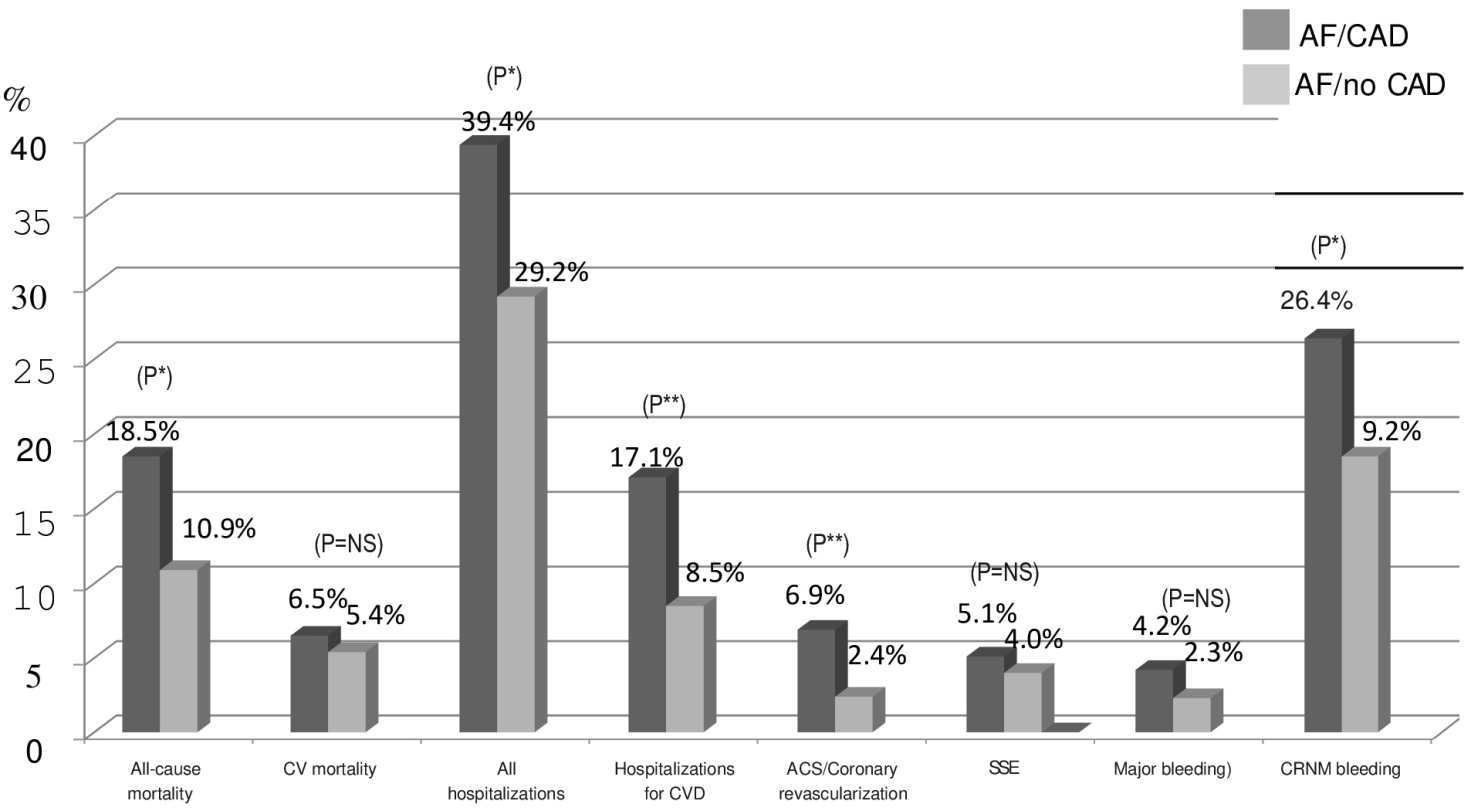
Major cardiovasular events at one year in atrial fibrillation patients with or without cornonary artery disease. AF: atrial fibrillation; CAD: coronary artery disease; CRNM: clinically relevant non-major (bleeding}; CVD: cardiovascular disease; SSE: stroke/systemic embolism. (*p ≤ .01; **p ≤ 0.001).

Figure 1 shows the one-year outcomes in both groups. The two major complications in patients with AF, SSE and major bleeding events, were not significantly different in both groups of patients with or without CAD, however more patients with AF/CAD had CRNM bleeding events, ACS and coronary revascularization than the AF/no CAD patients. Rates of hospitalizations, overall and for cardiovascular cause, and all-cause deaths were significantly higher in patients with AF/CAD than those with AF/no CAD. Events related to sepsis or viral infections, including COVID-19 infection, and their systemic complications in the whole cohort of patients, accounted for 38.5% (235 of 611) of all the hospitalizations and for 18.6% (44 of 237) of all deaths.

## Discussion

The current study is unique because it, for the first time, evaluated the intersection of AF and CAD in a Middle Eastern cohort. The principal findings are: (i) about one in ten patients with AF had concomitant CAD, (ii) the presence of CAD in patients with AF was associated with worse baseline clinical profiles and higher risk of future ACS, coronary revascularization, all-cause hospitalization and all-cause mortality. These findings are in agreement with those reported by studies from other regions which showed that AF patients were more prone to develop CAD than the general population and vice versa, and also showed that AF patients with CAD had worse outcome compared to those who did not have CAD [20,21]. The worse outcome observed in the group of patients with AF/CAD is attributed to the synergistic impact of the long-term adverse events associated with these two diseases, presence of coexisting comorbid diseases, and presence of confounding factors inherent to clinical registries.

The number of patients with AF is increasing on global and regional levels due to the aging of populations and increase in life expectancy. Atrial fibrillation, especially in the elderly, rarely exists as a single entity and is usually part of a wide range of cardiometabolic diseases, such as hypertension, CAD, diabetes mellitus and obesity. These conditions are associated with left atrial anatomical changes and/or electrical remodeling and subsequent persistence of AF [21,22,23]. In addition to sharing common risk factors, AF and CAD also interact with each other directly. Coronary artery disease, especially ACS, can potentially lead to the same pathophysiologic mechanisms that generate and sustain AF including reentry, focal ectopic activity, and neural remodeling [24]. On the other hand, AF is known to promote or exacerbate CAD by initiating atherosclerosis, thrombosis and embolism and causing mismatch of myocardial blood supply and oxygen consumption [25]. In the current study, the three classical cardiovascular risk factors (hypertension, diabetes mellitus and dyslipidemia) were present in the majority of all of the patients with AF, but specifically more prevalent in patients with concomitant CAD.

Acute and long-term management of AF patients who have CAD is challenging. Preventing stroke and systemic embolism due to AF necessitates the use of OACs, and prevention from ACS, coronary revascularization, stent thrombosis and cardiovascular death in CAD patients relies mainly on the use of long-term oral antiplatelet agents [7,8,26]. Hence, in the rather commonly encountered patient who has AF and CAD, the use of complex triple antithrombotic regimen exposes such patient to excess bleeding risks and calls for careful tailoring of the components of the pharmacotherapy and their duration in order to balance the risks of ischemic and bleeding events [27]. The majority of patients (8 of 10) in both groups in the current study received OACs, and more patients with AF/CAD received DOACs than VKA compared to the patient in the AF/no CAD group. Furthermore, seven of 10 patients in the AF/CAD group used single or double antiplatelet agents compared to only four of 10 patients in the AF/no CAD group. The available data from clinical studies and registries seem to favor dual therapy with a DOAC plus a P2Y12 inhibitor to prevent stroke, stent thrombosis and coronary ischemic events in AF patients who undergo percutaneous coronary intervention for ACS [28]. This strategy is advocated as a more reasonable and safe option than using triple therapy with VKA, aspirin and P2y12 inhibitor with respect to major bleeding events [26,27,28].

In this study, one-year outcomes of cardiovascular deaths, SSE and major bleeding events were not different between the groups of AF/CAD and AF/no CAD. However, all-cause mortality, all-cause hospitalization and hospitalization for cardiovascular reason, ACS, coronary revascularization and CRNM bleeding events were higher in the AF/CAD group than the AF/no CAD group. Not unexpectedly, mortality and morbidity occurred at higher rates among patients in the AF/CAD group than patients in the AF/no CAD group due to the higher prevalence of comorbid diseases such as HTN, diabetes mellitus, HF and CKD in the former group of patients. One important fact to be stressed is the unique timing of patient enrollment in the current study which took place during the COVID-19 pandemic with its related lockdown. This might explain the observed overall high mortality and morbidity. Nearly 1/3 of all hospitalizations and 12% of all deaths were related to the COVID-19 infection, sepsis and their complications. This is in concordance with findings by other studies that demonstrated a significant increase in cardiovascular disease mortality and morbidity during the COVID-19 pandemic compared with previous years [29].

Patients with AF and CAD suffered about three-fold higher incidence of overall major and non-major bleeding events compared to patients with AF but no CAD. This could be related to the utilization of antiplatelet agents and coronary revascularizations in higher number of AF/CAD patients than AF/no CAD patients [30]. Furthermore, the need for more coronary revascularizations in patients with AF and CAD than AF/no CAD adds further cardiovascular burden on this group patients with the inherent risk of the procedures on the short and long term follow up.

Several limitations should be acknowledged. Observational, non-interventional studies have inherently potential bias of residual confounding, incomplete data collection and potentially limited patient’s recall of events and complications. Recruiting consecutive patients could have overcome bias in enrollment. Furthermore, the fact that the major cardiovascular events evaluated in this study were hard endpoints, such as death, stroke, ACS, coronary revascularization and major bleeding, makes recall issues by patients very unlikely. We did not evaluate the outcomes according to the type of clinical syndromes associated with the diagnosis of CAD (i.e., ACS vs. chronic stable angina) because of the small numbers of patients in each of these two categories that might hamper a meaningful statistical analysis. The generalizability of the results of this to all countries in the region might be limited because our cohort was recruited from tertiary care centers and cardiology clinics in one country. Despite these limitations, this study adds an important scientific contribution to the contemporary knowledge concerning the important group of AF patients who have concomitant CAD who have not been studied previously in the Middle East.

## Conclusions

One in 10 Middle Eastern patients with AF had concomitant CAD. In addition to having a worse baseline clinical profile than those with AF but no CAD, patients with AF and CAD had worse one-year outcome. Optimal pharmacotherapy with OACs and antiplatelet agents and close follow up of this subgroup of AF patients should be practiced in order to mitigate the potential occurrence of future adverse cardiovascular events.

## Data Accessibility Statement

Readers can access the data supporting the conclusions of the study by contacting the corresponding author directly (a.hammoudeh@istisharihospital.com).
